# Growth after renal transplantation

**DOI:** 10.1007/s00467-008-0787-0

**Published:** 2009-07-01

**Authors:** Jérôme Harambat, Pierre Cochat

**Affiliations:** 1 Département de Pédiatrie and Inserm U820, Hôpital Edouard-Herriot and Université Claude-Bernard Lyon 1, Lyon, France; 2 Département de Pédiatrie, Unité de Néphrologie Pédiatrique, Hôpital Edouard Herriot, place d’Arsonval, 69437 Lyon, France

**Keywords:** Children, Corticosteroids, Growth, Growth hormone, Quality of life, Renal transplantation

## Abstract

Growth may be severely impaired in children with chronic renal insufficiency. Since short stature can have major consequences on quality of life and self-esteem, achieving a ‘normal’ height is a crucial issue for renal transplant recipients. However, despite successful renal transplantation, the final height attained by most recipients is not the calculated target height. Catch-up growth spurts post-transplantation are usually insufficient to compensate for the retardation in growth that has occurred during the pre-transplant period. Longitudinal growth post-transplantation is therefore influenced by the age at transplantation but also by subsequent allograft function and steroid exposure, both of which interfere with the growth hormone/insulin-like growth factor axis. The management of growth retardation in renal transplant recipients includes adequate nutritional intake, correction of metabolic acidosis, prevention of bone disease, steroid-sparing strategies and a supraphysiological dose of recombinant human growth hormone in selected cases.

## Introduction

One of the goals of renal transplantation in children is to restore an optimal quality of life (QOL), including the optimization of final height. However, catch-up growth post-transplantation is generally not sufficient to compensate for the deficit that has been acquired during the pre-transplant period. Growth retardation post-transplantation is multifactorial and associated with impaired medical and psychosocial outcomes. Despite numerous recent developments in pediatric renal transplantation, achieving an adequate final height remains a challenging issue for such recipients.

## Growth assessment

Growth assessment and management should be performed in any pediatric transplant recipient [1]. Anthropometric parameters, including height, body weight, body mass index (plus head circumference in children less than 3 years of age), should be monitored every 3 months in children less than 3 years of age, then every 6 months until final height is reached. Final height is reached as the growth velocity per year minus 1–2 cm after puberty has occurred. The target height (H, in cm) is based on mid-parental height (girls = [H_mother_ + H_father_–13]/2; boys = [H_mother_ + H_father_ + 13]/2) according to Tanner method [2]. A more recent formula based on parental height standard deviation score (SDS) and independent of sex has been proposed [3]. Growth parameters should be plotted on growth charts using either SDS or centiles adapted to gender and local standard measurements.

## Growth features post-transplantation

### Magnitude of growth impairment post-transplantation

Data from the North American Pediatric Renal Trials and Collaborative Studies (NAPRTCS) 2006 annual report reported that the mean height SDS was −1.4 in a cohort of over 1500 patients aged 19 years or more [4], with 25% of these patients having a height SDS of −2.3 or worse and 10% being below −3.3 SDS. During the last 10 years, single center reports have specifically addressed the issue of final height after renal transplantation in childhood [5–10]. The percentage of patients who achieved a normal final height (height SDS ≥ −1.88 , i.e. third centile) ranged from 42 to 75% (Table 1) which is higher than the that reported in previous studies in which 62–77% of patients exhibited growth failure at 18 years of age or more [11, 12].

**Table 1:** Summary of recent studies assessing final height in pediatric renal transplant recipients

Study	Tx period	Population (*n*)	rhGH post-Tx (*n*)	Age at Tx (years)	Follow-up (years)	Height SDS^a^			Growth determinants
						At Tx	Final Height	Normal height^b^	
Andre et al. 2003 [5]	1975	19 (11 boys)	0	13.2	10.9	−3.1	−2.6	42%	Height at ESRD; duration and cause of ESRD
Englund et al. 2003 [6]	1981–1994	24 (8 boys)	6	9.5	7.6	−1.7	−1.1	75%	Height at Tx; bone age; graft function
Ninik et al. 2002 [7]	1985–1998	82 (53 boys)	0	10.3	> 5	−2.1 (n = 82)	−1.3 (*n* = 47)	-	Height at Tx; age at Tx; final GFR; steroid dose
Nissel et al. 2004 [8]	1983–2002	37 (18 boys)	0	10.2 (girls)	8.5	−2.9 (girls)	−1.9 (girls)	68%	Height at Tx; graft function
				12.5 (boys)		−1.7 (boys)	−1.0 (boys)		
Offner et al. 1999 [9]	1970–1993	100 (47 boys)	7	13.6	13.1	−2.2	−2.4^c^	47%^c^	-
Rodriguez-Soriano et al. 2000 [10]	1986–1999	32 (17 boys)	3	12.1	7.2 (median)	−1.6 (girls)	−1.2 (girls)	69%	Height at Tx and at start of dialysis; duration of dialysis
						−1.4 (boys)	−1.6 (boys)		

*Tx* Transplantation, *ESRD* end stage renal disease, *GFR* glomerular filtration rate, *SDS* standard deviation score, *rhGH* recombinant human growth hormone

^a^Height values are expressed in SDS compared with local measurements

^b^Normal height is defined as a final height > − 2 SDS

^c^Final height in 84 non-cystinotic patients: −1.8 SDS (56% achieved normal height)

### Impact of growth failure post-transplantation

Growth delay post-transplantation may be associated with a worse medical outcome. Furth et al. [13] showed that children with end stage renal disease (ESRD) and moderate (−2 > SDS > −3) or severe (< −3 SDS) growth retardation had a significantly higher risk of death and hospitalization, even after adjustment for treatment modalities (dialysis or transplant). Such events were mainly attributed to infectious diseases, suggesting that growth failure might be a marker of poor nutritional status, a known risk factor for infectious complications.

Growth failure may also have an impact on psychological and social development, self-esteem and QOL. Behavioral and cognitive disorders, including immaturity, inhibition, anxiety, attention deficits and learning disability, have been reported in children with short stature [14, 15]. Such difficulties were attributed to family overprotection and negative social experience related to the child’s short stature, leading to impaired emotional and social development. Experience in patients with growth hormone deficiency or idiopathic short stature has shown that the use of recombinant human growth hormone (rhGH) improves behavior disturbances [16]. In adults who were transplanted during childhood, short stature has been associated with a lower marital status, a lower level of education and a lower level of employment [17]. Moreover, Rosenkranz et al. reported that more than one-third of adults with childhood-onset ESRD were dissatisfied with their body height [18]. Positive perception of QOL significantly correlated with satisfaction with adult height.

## Factors influencing growth post-transplantation

Growth after transplantation is mainly affected by the degree of pre-transplantation growth deficit, age at transplantation, graft function and exposure to glucocorticoids.

### Pre-transplantation growth

Growth failure is a common feature in children with chronic renal insufficiency (CRI). Growth determinants during the pre-transplant period may include age at diagnosis of CRI, nutritional intake, primary disease, tubular impairment, renal osteodystrophy, hormonal disorders and pre-transplantation use of steroids. Two periods are at particular risk of impaired growth velocity: infancy and puberty [19, 20]. Height velocity generally decreases when the glomerular filtration rate (GFR) is below 30 mL/min per 1.73 m^2^ [21]. However, there is evidence that growth impairment begins earlier. Indeed, in a subset of the NAPRTCS registry [4] that includes 1901 patients with moderate CRI (estimated GFR between 50 and 75 mL/min per 1.73 m^2^), 21.5% had standardized height measurement below the third centile (height SDS ≤−1.88) and in 2477 children with an estimated GFR of 25–50 mL/min per 1.73 m^2^, 36.8% had a height SDS of less than −1.88. Children enrolled in this registry are growth impaired at initiation of dialysis and exhibit a deceleration of growth during the following 6, 12 or 24 months—with the exception of children aged 0–1 years. At the time of transplantation, the mean height SDS measured in 8659 children was −1.86 for boys and −1.72 for girls [4].

Pre-transplantation growth impairment may be reduced by adequate conservative treatment of CRI, including aggressive nutritional support during infancy and early childhood [22] and treatment with rhGH when indicated [23]. This has resulted in a significant improvement of pre-transplantation growth; data from the NAPRTCS [4] has shown that height SDS at the time of initial transplant has improved from a mean of −2.4 in 1987 to −1.5 in the 2000 and 2003 cohorts.

### Role of age at transplantation

**Fig. 1:**
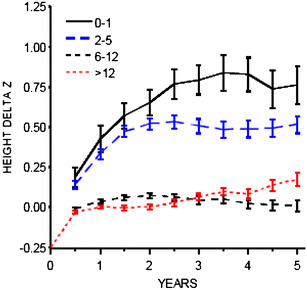
Mean changes (±SE) from baseline in height standard deviation score (SDS) by age at transplant (data from the NAPRTCS 2006 annual report)

In the NAPRTCS 2006 report [4], only infants and pre-school age children (2–5 years) exhibited catch-up growth post-transplantation (Fig. 1). However catch-up growth occurred mainly during the first 2 years post-transplantation, with no further improvement being observed thereafter; preschool-aged children who had the greatest deficit at transplant (−2.26 SDS) exhibited the best growth improvement of 0.5 SDS. Conversely, school age children (6–12 years), and adolescents demonstrated either no improvement or even a decrease in height SDS (Fig. 1). The prepubertal growth deceleration that occurs in the normal population is prolonged after transplantation, and puberty and bone age are usually delayed [8, 12]. This means that growth continues for longer than normal but height gain is rarely as much as expected due to loss of height potential [8, 24]. However, some authors have reported that significant catch-up growth can occur after transplantation even in children of pubertal age [25]; the authors speculated that catch-up growth may be related to the early use of a low-dose and alternate-day steroid regimen.

### Effect of graft function

The effect of a reduced GFR on growth has been known for a long time [26]. A report from the NAPRTCS that assessed final adult height in 237 subjects who received a transplant before 11 (girls) or 12 (boys) years of age showed that a decreased GFR was an independent predictor of reduced final height [27]. Guest et al. reported that prepubertal children with a serum creatinine level above 120 μmol/L did not exhibit catch-up growth during the first year post-transplantation [28]. More recently, Nissel et al. showed that prepubertal catch-up growth and total pubertal height gain correlated positively with GFR [8].

### Role of steroid therapy

Since the introduction of steroid therapy more than 50 years ago, it became clear that daily and prolonged steroid administration leads to growth impairment [29]. Steroid therapy inhibits growth by both interfering with the hypothalamus-pituitary/growth hormone/insulin-like growth factor axis and having a direct effect on bone formation (see below). Pharmacokinetic studies of methylprednisolone in pediatric liver and kidney transplant recipients have demonstrated that the area under the serum concentration–time curve (AUC) rather than dose was predictive for growth retardation [30, 31]. However, this correlation between AUC and growth was not unanimously found in transplanted children treated with prednisone or prednisolone [32].

### Other factors

Many other factors may contribute to post-transplant growth retardation.

#### Donor type

Pape et al. identified 51 boys who received a renal transplant (30 deceased donors and 21 living related donors) before the rhGH era, who were followed for at least 5 years [33]. Children who received a living donor graft had a significantly greater height SDS and growth velocity during the first 5 years post-transplantation than those who received a graft from a deceased donor. Interestingly, this difference remained significant after adjustment for potential confounders, including GFR. In the latest NAPRTCS report, donor source seemed to predict height SDS changes 2 years post-transplantation, but the mean difference was less than 0.3 SDS [2].

#### Pre-emptive transplantation

As dialysis is associated with decreased growth velocity, pre-emptive renal transplantation may optimize final height. Some authors reported better height SDS in the first years post-transplantation in those children who received a pre-emptive renal transplant compared to those with dialysis prior to transplantation [6, 25].

#### Race

A previous NAPRTCS report suggested that race may also have an impact on growth following transplantation [34]. The change in height SDS was negative in African-American and Hispanic recipients but positive in Caucasians. However, race did not significantly influence growth in multivariate analysis.

## Pathophysiology of growth impairment post-transplantation

### Disturbances in the growth hormone/insulin-like growth factor axis

In humans, longitudinal bone growth is achieved primarily by endochondral ossification, and an intact function of the somatotropic hormone axis is essential for normal growth. In healthy individuals, growth hormone (GH) is secreted by the pituitary gland in a pulsatile pattern; such a mechanism is regulated by GH releasing hormone (GHRH), GH release inhibiting hormone (GHRIH) and other feedback regulator agents. Growth hormone acts on the liver and other tissues, where it stimulates the synthesis of insulin-like growth factor-1 (IGF-1) and its binding proteins (IGFBP). Circulating free IGF-1 stimulates the proliferation of chondrocytes in the growth plate [35]. The IGFBPs, in turn, modulate IGF-1 activity, mostly by an inhibitory action mediated by IGFBP-3. Growth impairment in renal transplant recipients is multifactorial, and the somatotropic axis may be disturbed by several complex mechanisms.

*Glucocorticoids* are known to interfere with the GH/IGF axis by inducing down-regulation of GH receptors and inhibition of IGF-1 synthesis [36] and by modifying the equilibrium among IGFBP subtypes [37]. However, exogenous GH may reverse the catabolic and growth-depressing effects of glucocorticoids [38, 39]. In addition, glucocorticoid treatment directly affects growth plate function by suppressing chondrocyte proliferation, reducing bone formation and altering endochondral ossification [40, 41].

*Impaired renal function* in transplant recipients per se also contributes to growth disturbance so that even a minimal graft dysfunction may be associated with growth retardation [42]. Due to both decreased metabolic clearance and altered release pattern, GH concentration is normal or elevated in any child with CRI [43, 44] so that such patients exhibit a resistance profile to GH action. Indeed, it has been shown that uremia results in reduced GH receptor density and in a defect of intracellular GH receptor signal transduction, so that IGF-1 transcription is decreased [45]. Furthermore, modifications in IGFBPs levels limit the bioavailability of free IGF-1 [46, 47].

### Metabolic acidosis

The presence of metabolic acidosis may additionally affect growth through a reduction of GH and IGF-1 secretion [48] as well as by GH resistance [49]. This phenomenon is attributable to a down-regulation of hepatic IGF-1 and GH receptor expression and increased expression of IGFBP-2 and -4, both of which inhibit the effects of IGF-1 [50]. In addition, metabolic acidosis has been shown to have an inhibitory effect on cartilage cell progression and endochondral bone formation in experimental studies [51]. It also leads to reduced albumin synthesis, increased calcium efflux from bone and protein degradation. However, there is no evidence that a correction of metabolic acidosis will have a beneficial effect on growth post-transplantation.

### Secondary hyperparathyroidism

Long-term secondary hyperparathyroidism may persist after renal transplantation [52] and have a negative impact on bone turnover and growth plate function [48]; this may be attributable to abnormal epiphyseal growth plate due to reduced PTH/PTHrP (PTH-related peptide) receptor and type X collagen expression. A potential benefit of treating for secondary hyperparathyroidism is an improvement in linear growth, although there is no strong evidence supporting this.

### Nutrition

Independently of the above mechanisms, proteinocaloric deficit and subsequent malnutrition result in growth retardation, mainly in infants and young children. Indeed, protein restriction leads to a resistance to GH action at the hepatic level, an increase in IGF-1 clearance rate and, consequently, a reduction of IGF-1 levels [53–55].

### Sex hormones

Disturbances of the gonadotropic axis may contribute to altered growth patterns, with a delay and a shorter duration in the pubertal growth spurt [8, 12, 56].

## Management of growth post-transplantation

### Conservative strategies

Growth velocity can be improved by conservative approaches, including adequate nutritional intake, correction of metabolic disorders, prevention of renal osteodystrophy and steroid-sparing protocols (Table 2). The use of alternate-day steroids in kidney transplantation was first described in the 1970s [57], and several studies carried out subsequently have demonstrated an improvement in growth in patients on daily low or alternate-day steroid therapy [58, 59]. The use of deflazacort, a synthetic glucocorticoid derived from prednisolone, has led to improved growth velocity with a comparable immunosuppressive effect [60], but there are few data on this drug because of its limited availability. Encouraging results have been reported with steroid avoidance or early withdrawal protocols [61–64], but additional evidence is expected from ongoing trials.

**Table 2:** Conservative methods to optimize growth velocity post-transplantation [1, 87]

Causes of growth impairment	Methods
Reduced GFR	Prevention and management of chronic allograft nephropathy
	Treatment of acute rejection episodes
	Drug compliance
Steroid therapy	Daily low or alternate day steroid therapy
	Steroid avoidance/withdrawal under evaluation
Bone disease	Target PTH within normal range in CKD stage 2–3 and < 2× upper limit in CKD stage 4
	Target plasma phosphate within age-appropriate normal range
Metabolic acidosis	Target plasma bicarbonate > 22 mmol/L
Malnutrition	Adequate nutritional intake
Comorbidities	Assessment and control of comorbidities which may impair growth (chronic inflammation, liver, lung or heart diseases)

*PTH* Parathyroid hormone; *CKD* chronic kidney disease

### Growth hormone therapy

#### Efficacy and safety

The rationales for the use of rhGH in short children with renal transplants are: (1) exogenous GH can be considered to be a substitutive therapy in children with glucocorticoid-induced GH hyposecretion and (2) rhGH may restore IGF bioactivity in those with normal GH secretion but decreased IGF bioavailability [65, 66]. Four randomized controlled trials have been conducted to evaluate the safety and efficacy of rhGH after renal transplantation [67–70]. All four studies have shown a significant improvement in growth velocity in the treated group compared to the controls (Table 3), but none of these trials has remained controlled for more than 1 year. Although previous observations have suggested an increased risk of acute rejection [71] and controversial effects on GFR [72, 73], none of the randomized controlled trials have demonstrated an increased incidence of acute rejection or a change in GFR. However, two of them found that a history of two or more acute rejection episodes before the initiation of rhGH was predictive of a subsequent rejection episode following the initiation of rhGH therapy [69, 70]. More recently, a non-randomized study from the NAPRTCS compared 513 rhGH-treated transplant recipients to 2263 untreated children over a 5-year period [74]. The authors concluded that children younger than 10 years of age grew better than older ones; the final height was superior in the rhGH-treated group (mean cumulative increase of 3.6 cm), without any significant change in allograft function and graft failure rate, and the incidence of adverse events was similar in both groups.

**Table 3:** Summary of randomized controlled trials of rhGH therapy in children after renal transplantation

Study	Design	Population	Growth velocity (cm/year)		Safety	
			rhGH group	Controls	Rejections	GFR
Hokken-Koleaga et al. 1996 [67]	6-months crossover	*n* = 11			No episode	No change
		5 prepubertal	3.9 (6 months)	1.0		
		6 pubertal	5.3	1.5		
Maxwell et al. 1998 [68]	1-year randomized	*n* = 22			8/13 in rhGH group vs. 5/9 in controls^a^	No change
		15 prepubertal	8.1	3.7		
		7 pubertal	10.1	3.9		
Guest et al. 1998 [69]	1-year randomized	*n* = 90	7.7	4.6	9/44 in rhGH group vs. 4/46 in controls^a^	No change
		55 prepubertal				
		35 pubertal				
Fine et al. 2002 [70]	1-year randomized	*n* = 63	9.0	4.2	None in rhGH group vs. 3 in controls	No change in SCr^b^
		40 prepubertal				
		23 pubertal				

^a^Not significant

^b^SCr, Serum creatinine

The immune system may be adversely affected by the GH-induced stimulation of cytotoxic T cells in vitro [75]. However, data in pediatric renal transplant recipients suggest either a transient or a moderate impact of rhGH on the immune system [76, 77].

A major concern with the use of rhGH is the potential risk of malignancy. There is no evidence of increased risk of lymphoproliferative disease with the use of rhGH post-transplant only. However, a significant association has been found between the use of rhGH during the pre-transplant period and the development of post-transplant lymphoproliferative disease [78]. Tydén et al. reported the development of renal cancer in the transplant kidneys of two adolescents treated with rhGH [79]. However, an analysis of data from companies commercializing rhGH was unable to identify rhGH as a risk factor for post-transplant renal cancer [80].

Other side effects have been reported under rhGH therapy, but they do not represent a significant risk and should not limit the current use of rhGH in growth-retarded transplanted children. Several studies have shown a transient elevation of glucose and insulin secretion in rhGH-treated patients with CRI or after renal transplantation [81]. There has been no report of diabetes mellitus development under rhGH, but one patient with cystinosis has been reported. Recombinant human growth hormone has also been found to induce an increase in lipoprotein(a) serum concentration without a significant change in cholesterol or triglycerides serum concentrations [82]. This increase did not persist over time after a long-lasting treatment with rhGH in patients with Turner syndrome [83]. Transient increases in alkaline phosphatase activity, serum phosphate concentration [70] or even PTH concentration [68] have been observed in randomized controlled trials, but rhGH did not induce an increased incidence of renal osteodystrophy; however, overt renal osteodystrophy may blunt the response to rhGH [84].

In a retrospective survey, benign intracranial hypertension was detected in about 1% of 1670 children with CRI [85]. This complication may be exacerbated by the presence of fluid overload and arterial hypertension associated with CRI.

#### Current recommendations

The use of rhGH in growth-retarded renal transplant recipients has not been approved by North American or European drug regulatory agencies, so that there are no clear guidelines for its use in such patients. In addition, the American Association of Clinical Endocrinologists does not recommend the use of rhGH after renal transplantation, unless it is given as a part of a research study [86]. However, based on published trials and registries, a recent report from the Kidney Foundation on Kidney Disease Improving Global Outcomes (KDIGO) has provided recommendations for its use after pediatric renal transplantation [1]. All growth parameters should be assessed and corrected before rhGH is initiated. Therapy with RhGH can be started when height falls below the third centile for age and sex. It remains uncertain whether GH therapy should be considered in children with still-normal relative height but low growth velocity. The recommended dose is 0.05 mg/kg per day (1.4 mg/m^2^ per day) in prepubertal children. In pediatric transplant patients with chronic kidney disease stages 2–4, bone disease should be managed according to K/DOQI guidelines [87]. A practical approach for the use of rhGH after transplantation is proposed in Table 4.

**Table 4:** Proposed recommendations for the use of rhGH in children with a renal transplant

Recombinant human growth hormone therapy	Factors to take into consideration
Target population for rhGH therapy	Prepubertal children
	Pubertal children?
	−2 SDS for height
	Poor growth velocity?
	Growth potential documented by open epiphyses
	Correction of other factors contributing to growth failure
Contra-indication	Active malignancy
Baseline evaluation	Pubertal stage
	Anthropometric assessment
	Target height
	Hip X-ray and bone age
	Fundoscopic examination
	Serum phosphate, calcium and PTH
Optimal dose of rhGH	0.05 mg/kg per day (4 IU/m^2^ per day)
Mode of administration	Daily subcutaneous injection
Follow-up evaluation	Height, weight, growth velocity every 3 months
	Close monitoring of graft function in children with a history of acute rejection
	Serum calcium, phosphate and PTH every 3 months
	Bone age every year according to growth profile
rhGH discontinuation	Achieved height endpoint
	Closed epiphyses
	Slipped femoral epiphyses
	Severe hyperparathyroidism
	Active malignancy
	Documented benign intracranial hypertension
	Non compliance

In summary, steroid-sparing/avoidance may be the first-line management of short transplant children with a good GFR (i.e. > 60 mL/min per 1.73 m^2^), whereas rhGH treatment may be proposed to those short patients with either impaired GFR (i.e. <60 mL/min per 1.73 m^2^) or the failure of steroid-sparing/avoidance despite a good GFR.

## Conclusion

Optimal growth is a major issue regarding post-transplantation quality of life and self esteem in children. The current final height of transplant patients has remained suboptimal, but recent strategies, including steroid-sparing/avoidance regimes, could further improve longitudinal growth in renal transplant children.

## Questions

(Answers appear following the reference list)

1. What has been learned from epidemiological data about post-transplant growth?
  - Most renal transplant children attain target height
  - Growth velocity increases after 3 years post-transplantation
  - Height SDS at transplantation has improved over time
  - Catch-up growth is primarily exhibited by adolescents
  - Quality of life of transplant recipients is not influenced by height
2. Which factor positively influences growth post-transplantation?
  - Male gender
  - Young age at transplantation
  - Primary tubular disorder
  - Prolonged steroid therapy
  - Dialysis prior to transplantation
3. Which mechanism contributes to glucocorticoids-induced growth retardation?
  - Decreased serum IGF-1 concentration
  - Reduced sensitivity to endogenous GH and IGF-1
  - Direct stimulation of chondrocytes proliferation
  - Decreased production of all IGFBPs
  - Disturbances in the gonadotropic hormone axis
4. How can growth potential be optimized?
  - Correction of metabolic acidosis
  - Adequate nutritional intake
  - Prevention of bone disease
  - Supraphysiological dose of rhGH when indicated
  - All of the above
5. Which of the following statements about the use of rhGH is correct?
  - The optimal dosage of rhGH is 0.02 mg/kg per day
  - There is evidence that rhGH improves final height
  - Randomized controlled trials with rhGH have shown an increased risk of acute rejection
  - rhGH may induce an elevation of serum insulin concentration
  - rhGH has been identified as a risk factor for post transplantation malignancy
